# Change in hippocampal theta oscillation associated with multiple lever presses in a bimanual two-lever choice task for robot control in rats

**DOI:** 10.1371/journal.pone.0192593

**Published:** 2018-02-12

**Authors:** Norifumi Tanaka, Katsunari Sano, Md Ashrafur Rahman, Ryota Miyata, Genci Capi, Shigenori Kawahara

**Affiliations:** 1 Graduate School of Innovative Life Science, University of Toyama, Toyama-shi, Toyama-ken, Japan; 2 Graduate School of Science and Engineering, University of Toyama, Toyama-shi, Toyama-ken, Japan; 3 Department of Mechanical Systems Engineering, University of Ryukyus, Okinawa-ken, Japan; 4 Department of Electrical and Electronic System Engineering, University of Toyama, Toyama-shi, Toyama-ken, Japan; Tokai University, JAPAN

## Abstract

Hippocampal theta oscillations have been implicated in working memory and attentional process, which might be useful for the brain-machine interface (BMI). To further elucidate the properties of the hippocampal theta oscillations that can be used in BMI, we investigated hippocampal theta oscillations during a two-lever choice task. During the task body-restrained rats were trained with a food reward to move an e-puck robot towards them by pressing the correct lever, ipsilateral to the robot several times, using the ipsilateral forelimb. The robot carried food and moved along a semicircle track set in front of the rat. We demonstrated that the power of hippocampal theta oscillations gradually increased during a 6-s preparatory period before the start of multiple lever pressing, irrespective of whether the correct lever choice or forelimb side were used. In addition, there was a significant difference in the theta power after the first choice, between correct and incorrect trials. During the correct trials the theta power was highest during the first lever-releasing period, whereas in the incorrect trials it occurred during the second correct lever-pressing period. We also analyzed the hippocampal theta oscillations at the termination of multiple lever pressing during the correct trials. Irrespective of whether the correct forelimb side was used, the power of hippocampal theta oscillations gradually decreased with the termination of multiple lever pressing. The frequency of theta oscillation also demonstrated an increase and decrease, before and after multiple lever pressing, respectively. There was a transient increase in frequency after the first lever press during the incorrect trials, while no such increase was observed during the correct trials. These results suggested that hippocampal theta oscillations reflect some aspects of preparatory and cognitive neural activities during the robot controlling task, which could be used for BMI.

## Introduction

The hippocampal theta oscillation in animals has been implicated in a variety of cognitive processes [1], including the decision-making process [2–4]. Further, there is a change in the state of hippocampal theta oscillations prior to the alteration in behavior or motor output [5–7], which if integrated into the brain-machine interface (BMI) could be a cue for attention to prepare the output device for incoming motor commands. Consistent with this idea, there have been several human studies that have analyzed pre-movement cortical oscillatory activities [8] and utilized activities to decode the intention of limb movement [9, 10]. In addition, the theta oscillation recorded from the frontal midline area in humans (FM theta) has also been implicated in attention-related activities during various mental tasks [11]. Since there are functional similarities between the rat hippocampal theta and human FM theta oscillations [12], it is useful to investigate the properties of hippocampal theta oscillation in rats during a task that is similar to that used in BMI development, such as a visually guided operant task.

In a previous study, we attempted to estimate the movement of an e-pack robot, which was controlled by the rat with lever pressing, from the analysis of the local field potential (LFP) of the motor areas [13]. During the task, the rat pressed one of the two levers several times with the ipsilateral forelimb to bring the food-carrying robot to front of the rat. Therefore, this task was more complicated and cognitively demanding than a standard operant lever press task, during which a rat pressed the lever only once with its dominant forelimb to obtain a reward. The contribution of the hippocampus to this task may be increased as the task becomes more difficult, as reported in other tasks [14–19]. For example, in a two-lever choice task, analysis of metabolic activity revealed a role for the hippocampus in effort-based decision making [18]. Further, hippocampal lesions selectively impaired an aspect of higher cognition [19]. In addition to the task difficulty, the bimanual use in the present task might also elevate cognitive demand. It has been reported that rats show handedness, i.e., a preference to use the right forepaw [20], and that reversal of handedness activates the hippocampus [21]. The dependency of hippocampal theta oscillations on task difficulty is like that reported in FM theta [22]. Therefore, the bimanual robot manipulation task is a useful approach to evaluate hippocampal/FM theta oscillations as the pre-movement cue in BMI, together with the role of hippocampal theta oscillation in a cognitively demanding task.

In the present study, we investigated hippocampal theta oscillations during the bimanual two-lever choice task for robot control, focusing on the preparatory phase to elucidate the properties of the hippocampal theta oscillation, which could be used in BMI. We demonstrated that the relative power and dominant frequency of theta oscillation increased before the start of multiple lever pressing, irrespective of the whether the choice or used forelimb side was correct, suggesting the participation of hippocampal theta oscillations during the preparatory process for multiple lever pressing, but not during the cognitive process for the correct side or the detail of forelimb control.

## Materials and methods

### Animals and ethics statement

Male Wistar/ST rats that were 10 weeks old at the start of experiment (n = 5, Japan SLC, Inc., Hamamatsu, Shizuoka, Japan) were used for the current study. The rats were housed individually in standard plastic cages, on a 12/12 h light/dark cycle, with free access to drinking water. They were habituated to handling and their body weight was gradually reduced to approximately 85% over a week before training with food rewards was initiated. All experimental procedures were conducted in accordance with the NIH Guide for the Care and Use of Laboratory Animals and approved by the Experimental Animal Committee of the University of Toyama (A2014ENG-7). Throughout the experiment, all efforts were made to minimize the use of animals and to optimize their comfort.

### Conditioning procedures

**Fig 1:**
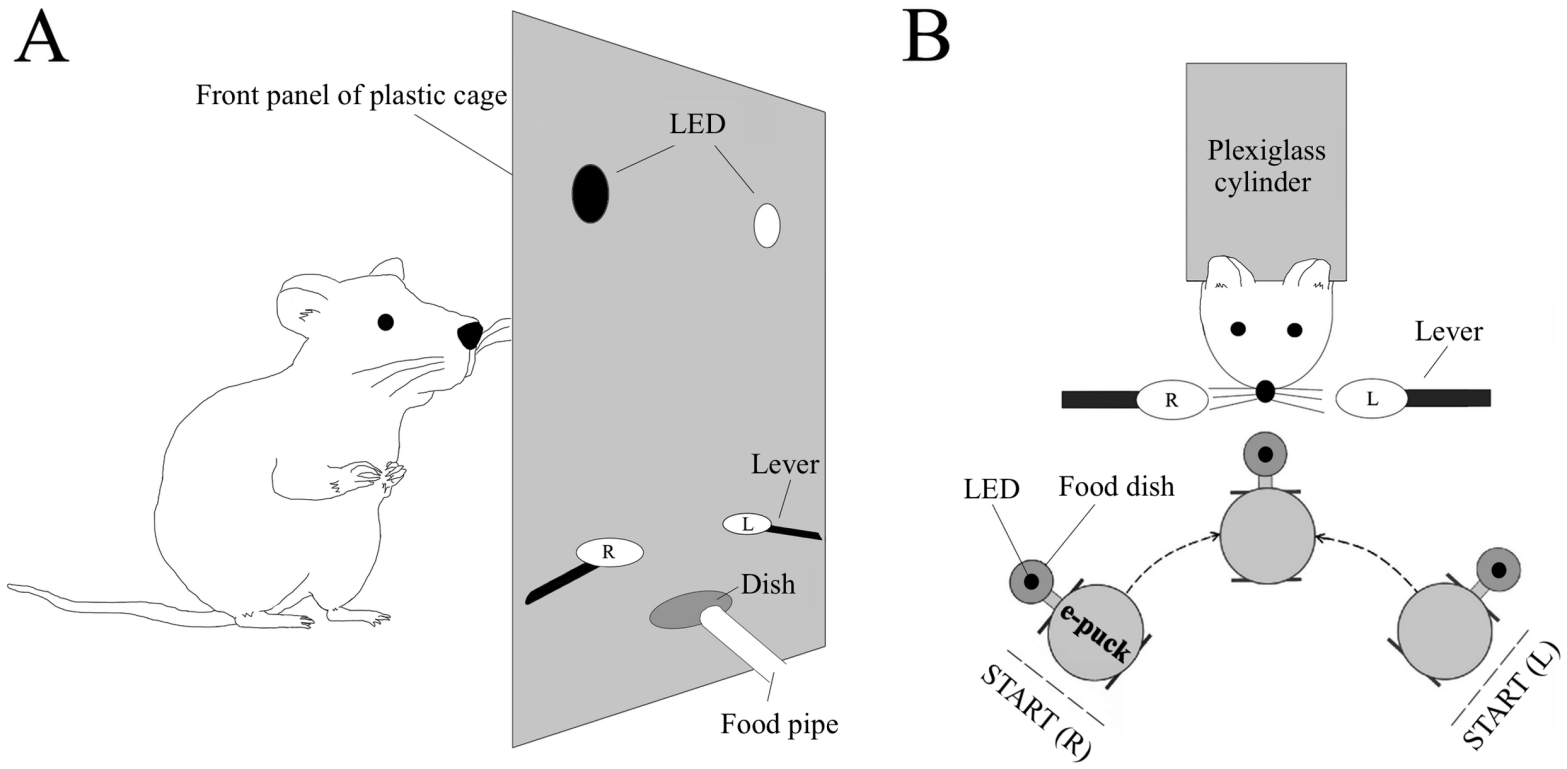
Conditioning apparatuses. (A) Schematic diagram of the operant lever press task in the first stage of conditioning (side view). Rats were trained to press one of the levers ipsilateral to the illuminating LED to receive food. The right (R) and left (L) levers were placed in the conditioning box and attached to micro-switches at their bases. (B) Schematic diagram of the robot controlling task in the second and the third stages of conditioning (top view). Restrained rats were trained to press the correct lever ipsilateral to the robot several times until the robot came to the area within their reach. The robot had a dish with food and an illuminating LED.

The rats were trained to press a correct lever to move a food-carrying robot towards them using a food reward and three progressive stages, which took approximately 3–4 weeks in total. A daily session contained approximately more than 20 trials; the rats received a small amount of food during each trial. A daily session was terminated if the rat stopped to press the lever or the session exceeded 1 h. At the end of daily session, they were fed additional food to maintain the body weight. During the first stage, a rat was placed in a transparent plastic cage (28×45×20 cm) equipped with two micro-switch levers, whose tips were set at a height of 0.5 cm and 7.5 cm apart horizontally, and two green light-emitting diodes (LED) set above the lever tips at 3.2 cm height. One of the LEDs was manually turned on at a time (Fig 1A). The rat was allowed to use either forelimb to press the correct lever beneath the illuminating LED to receive a food pellet. The side of illuminating LED was changed from session to session in such a way that the same side was not used in more than two consecutive sessions. Further, the total number of sessions was almost balanced between the right and left side. The rats were trained until they achieved more than 90% correct for both sides, calculated by dividing the number of correct lever presses by the total number of lever presses in a session. During the second stage of training, they were habituated to take food under a mild restrained condition with a neck collar and acryl cylinder, which limited the body movement and forelimb usage (Fig 1B). After two days of habituation, they were trained to press the lever ipsilateral to the position of e-puck robot (Cyberbotics Ltd., Switzerland) using only the ipsilateral forelimb. The robot had a food handler and green LED, which was constantly illuminated during a daily session. The robot was placed either on the right or left start point on a semicircle track, approximately 13 cm away from the reach of the rat, and moved towards or away from the rat along the track when the rat pressed the ipsilateral correct or contralateral incorrect lever, respectively [13]. The rat repeatedly pressed the lever until the robot came to the area within their reach. After the rat took the food, the robot was manually returned to the right or left start point. The micro-switch levers were connected through a serial interface to the computer, which controlled the e-puck robot via a Bluetooth interface. The side of the start point was identical throughout the trials in a daily session and was changed randomly from session to session as in the first stage. A trial was categorized as correct if the first and subsequent lever presses were ipsilateral to the robot, while it was categorized as incorrect if the first press was contralateral. Even during the incorrect trials, most of subsequent lever presses were correct. The rats were trained until they were able to achieve more than 90% correct for both sides, which was calculated by dividing the number of correct trials by the total number of trials. During the last stage, the start point side was changed randomly within a session and the rats were trained accordingly as described above.

### Surgery

After reaching 90% correct during the third stage, the rats underwent surgery to set the electrodes to record the hippocampal oscillation. The electrodes were constructed from a pair of twisted Teflon-coated stainless-steel wires (140 μm in diameter, A-M Systems, Sequim, WA, USA) and soldered to a pin header connector. The electrodes were implanted bilaterally in the dorsal hippocampus under anesthesia with sodium pentobarbital (65 mg/kg i.p., Kyoritsu Seiyaku, Tokyo, Japan). Isoflurane (1–2%, Abbot Japan, Osaka) was also used when necessary. The stereotaxic coordinates of electrode placements were 4.8 mm anteroposteriorly, 3.2 mm laterally, and 2.2 mm dorsoventrally from bregma, according to the standard brain atlas [23]. The final depth of electrode was decided based on the LFP profile recorded during the implantation. The connector was secured on the skull by acrylic dental cement and three stainless-steel screws, one of which was used as a ground electrode. After the surgery, the animals were injected with ampicillin (100 mg/kg i.p., Meiji Seika, Tokyo, Japan) and warmed until they moved spontaneously. During the recovery period, rats were fed wet paste food instead of standard pellet food.

### Analysis of the hippocampal oscillation

After a week of recovery, the same conditioning as in the third stage was restarted. The hippocampal LFP was recorded using the Cheetah system (Neuralynx, Tucson, AZ, USA). The hippocampal LFP was band-pass filtered between 1 and 475 Hz and sampled at 7,575 Hz, which was reduced offline to 1,515 Hz before further analyses. The data collected from three sessions in each rat were analyzed with custom-written programs on MATLAB (The MathWorks, Natick, MA, USA).

**Fig 2:**
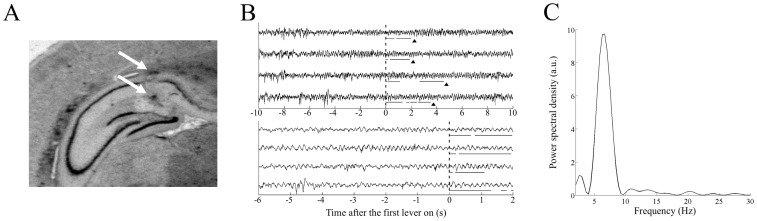
Electrode position and the hippocampal local field potential (LFP). (A) Typical electrode position in the dorsal hippocampus. The arrows indicate the tips of the twisted electrode, which were marked electrically after the completion of behavioral experiment. (B) Typical hippocampal LFP data around the lever press. The LFP data in four trials are shown on a longer and shorter time scale in upper and lower traces, respectively. The times before the first lever press are indicated as negative values. Horizontal lines under each LFP trace indicate the lever presses. The rat took a food from the robot after the final lever off, indicated by arrowheads in the upper traces. (C) Typical power spectral density of the hippocampal LFP during a session, plotted in arbitrary units.

**Fig 3:**
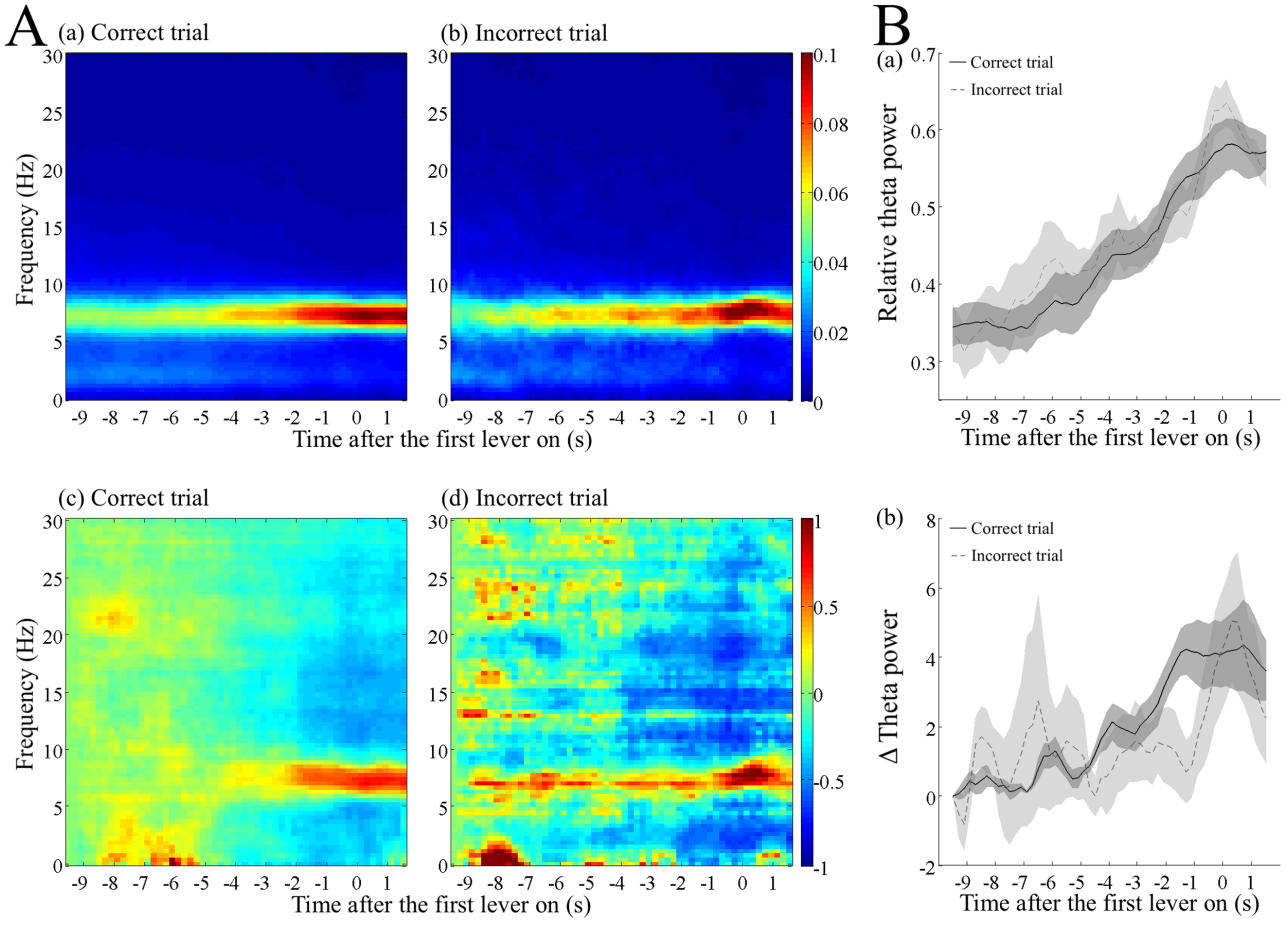
Increase in the relative and absolute theta power before the first lever press in the correct and incorrect trials. (A) Dynamic power spectra in the correct (a, c) and incorrect trials (b, d). A time window of 1 s and a stepping width of 0.2 s were used. The frequency resolution was approximately 0.36 Hz. The data were averaged over the trial type in each rat and then over all the rats. The relative power divided by the total power of 1–30 Hz (a, b) and the absolute power subtracted and normalized by that 10 s before the first lever press (c, d) were calculated for each frequency. The times before the first lever press are indicated as negative values. The pseudocolor scales in the right indicate the relative power and the normalized absolute power. (B) Increase in the relative (a) and normalized absolute (b) power of theta frequency band. The power data of each frequency in A were integrated over the theta frequency band of 6–9 Hz. The black and dotted lines indicate the data averaged over the correct and incorrect trials in each rat, respectively, and then over the rats. The shaded areas associated with the lines are standard error of the mean.

**Fig 4:**
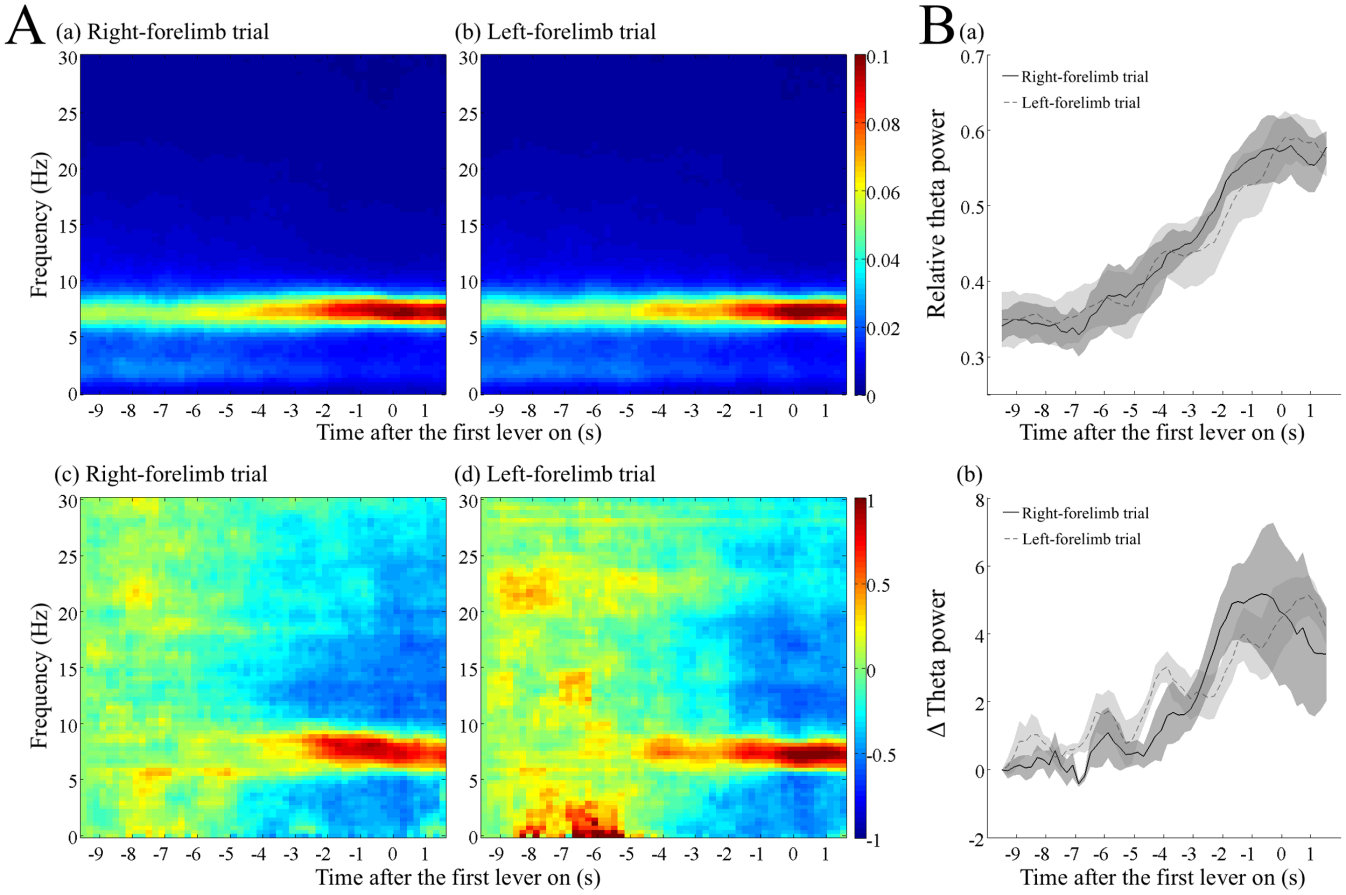
Increase in the relative and absolute theta power before the first lever press in the right- and left-forelimb trials. (A) Dynamic power spectra before the first lever press with the right (a, c) or the left (b, d) forelimb in correct trials. The parameters used for the analysis were the same as in Fig 3. The relative power (a, b) and normalized absolute power (c, d) of each frequency were calculated as in Fig 3. (B) Increase in the relative (a) and normalized absolute (b) power of theta frequency band (6–9 Hz). The black and dotted lines indicate the data averaged over the right- and left-forelimb trials, respectively, and then over the rats. The shaded areas associated with the lines are standard error of the mean.

**Fig 5:**
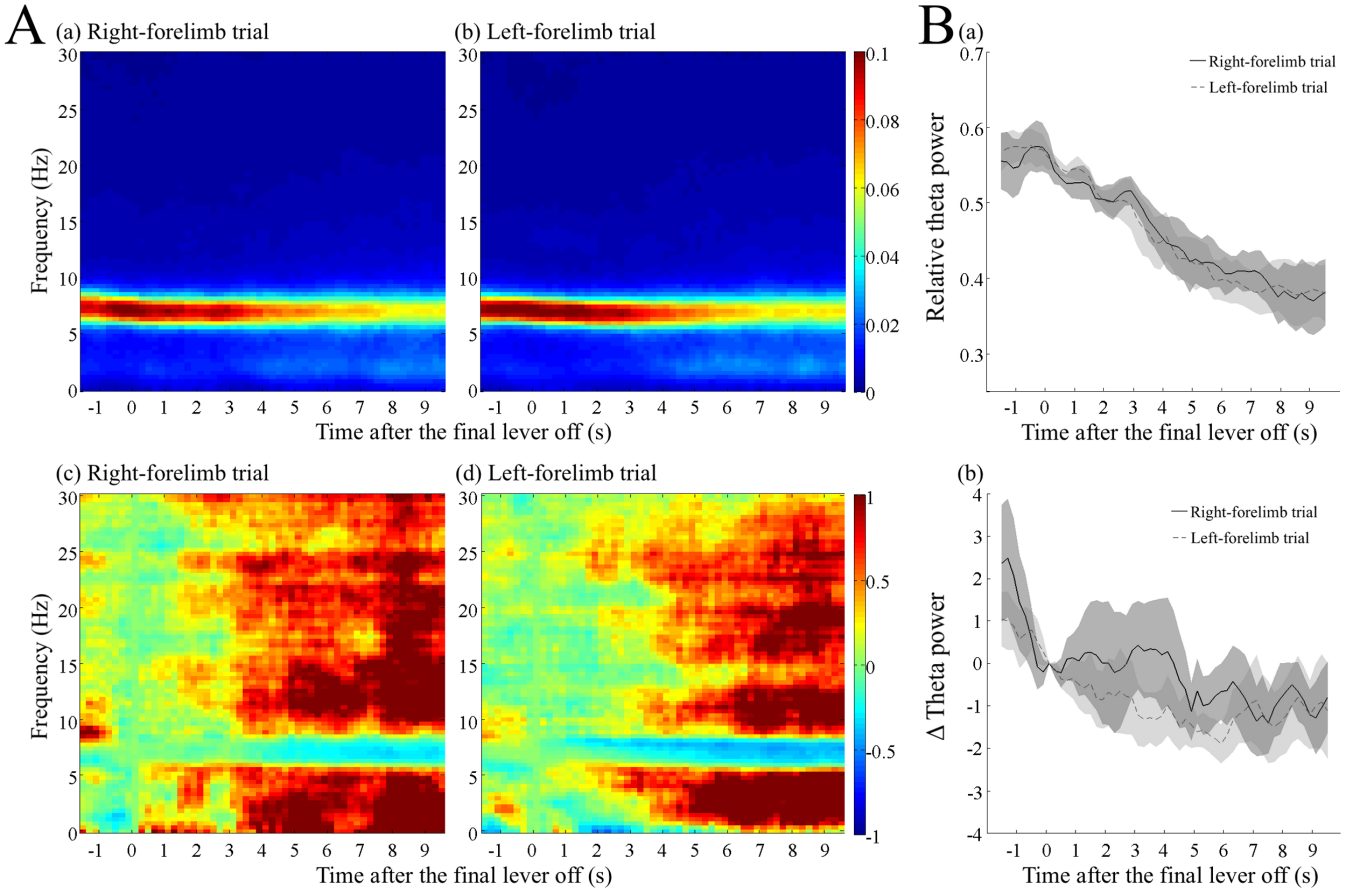
Decrease in the relative and absolute theta power after the termination of multiple lever pressing in the right- and left-forelimb trials. (A) Dynamic power spectra after the final lever off, with the right (a, c) or left (b, d) forelimb in correct trials. The parameters used for the analysis were the same as in Fig 3. The relative power divided by the total power of 1–30 Hz (a, b) and the absolute power subtracted and normalized by that at the time of lever off (c, d), were calculated for each frequency. The times before the final lever off are indicated as negative values. (B) Decrease in the relative (a) and normalized absolute (b) power of the theta frequency band (6–9 Hz). The black and dotted lines indicate the data averaged over the right- and left-forelimb trials in each rat, respectively, and then over the rats. The shaded areas associated with the lines are the standard error of the mean.

The analysis of the theta oscillations were conducted after confirming of the location of the electrode tips and spectral distribution during a sessions (Fig 2). To further analyze theta oscillations in the time before and after the multiple lever pressing (Figs 3, 4 and 5), we used the multi-taper FFT MATLAB package written by Mitra and Pesaran (1999) [24], with a frequency range of 1–30 Hz, a time bandwidth product of 1, 2 Slepian taper functions, a time-window of 1 s, and a stepping width of 0.2 s. The relative power of the theta frequency band (6–9 Hz) divided by the power of total band (1–30 Hz). In addition, the absolute power expressed as a fractional change of the 10 s before the first lever on (Figs 3 and 4) or that at the end of the final lever off (Fig 5). During each frequency, the fractions in each time-period were divided by the fractions of 10 s before the first lever on or 0 s in the final lever off to estimate the absolute change of power from those time periods, such as during the pre- or post-lever period. These analyzed data during a session were grouped into correct and incorrect trials. The data from correct trials were further divided into the right- and left-forelimb trials.

**Fig 6:**
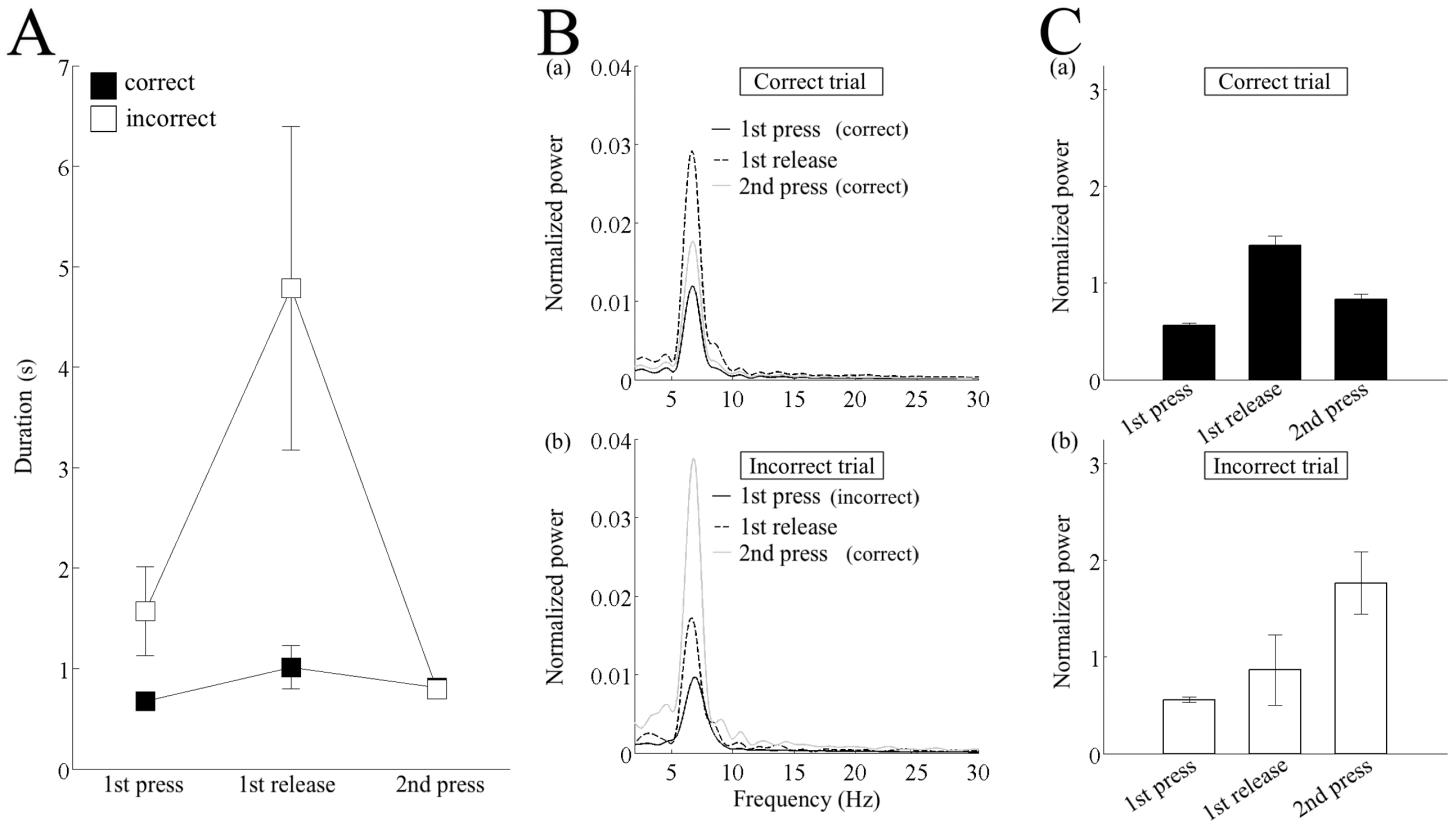
Differences between the correct and incorrect trials during the first and the second lever presses. (A) Duration of the time between the first lever-on and first lever-off (1st press), between the first lever-off and second lever-on (1st release), and between the second lever-on and second lever-off (2nd press). The data were averaged over the trial type in each rat and then over all the rats. (B) Absolute power spectral density normalized by the total power of the first press (see the Methods for details) in the correct (a) and incorrect trials (b). The spectrum was averaged over the trial type in each rat, and then over all the rats. (C) Normalized power of theta frequency band (6–9 Hz) in the correct (a) and incorrect trials (b). The error-bars indicate the standard error of the mean.

To analyze the change in theta power during the first and second lever presses as well as between them (Fig 6B), we calculated the power spectral density after matching the frequency resolution, which showed a variation due to the difference in data length. The power spectral density at each frequency was normalized by the total amount of those between 1 and 30 Hz during the first lever press, and then averaged over the trials and animals.

**Fig 7:**
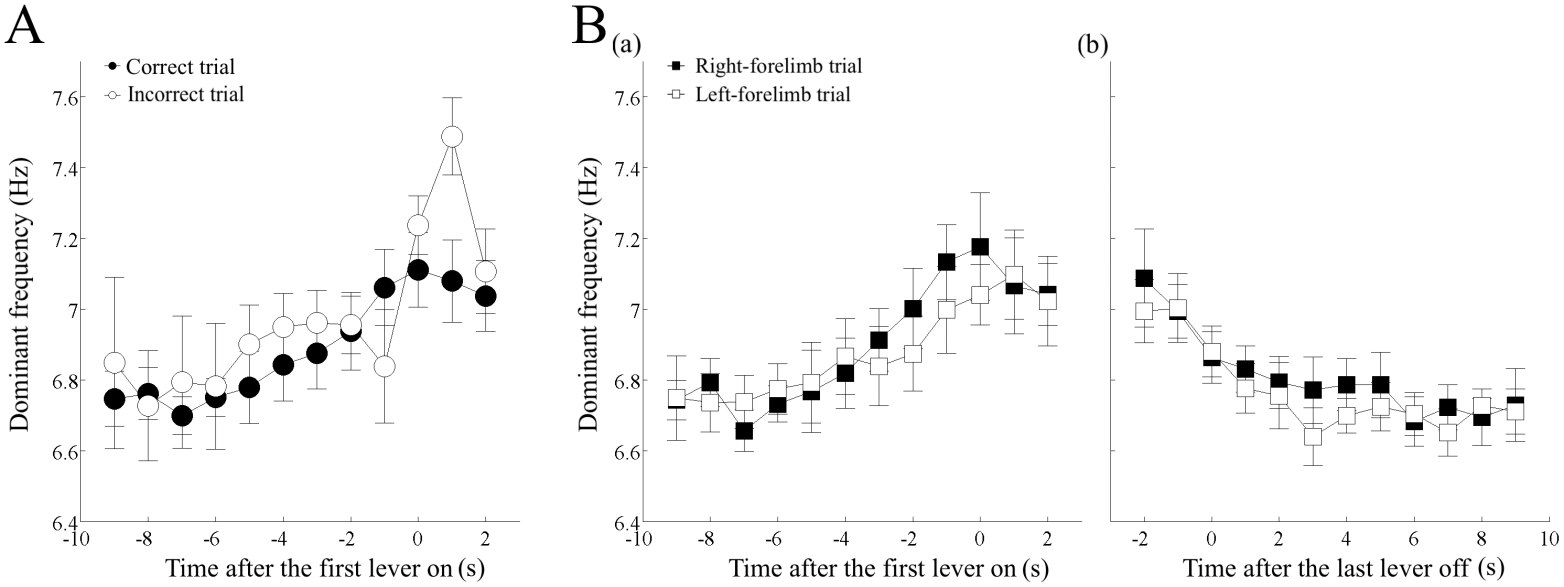
Change in the theta frequency before the first lever press and after the last lever release. (A) Increase in the dominant frequency of theta oscillation before the first lever press in the correct and incorrect trials. The filled and open circles indicate the data averaged over the correct and incorrect trials in each rat, respectively, and then over all the rats. (B) Increase in the dominant frequency of theta oscillation before the first lever press (a) and decrease after the final lever off (b) in the right and left forelimb trials. The filled and open squares indicate the data averaged over the right and left trials in each rat, respectively, and then over all the rats. The error-bars indicate the standard error of the mean.

The analysis of the theta frequency from the power spectrum was estimated as the weighted-average frequency of theta band (4–9 Hz) (Fig 7). This analyzed frequency data were also grouped into correct and incorrect trials. The data from correct trials were further divided as we described in the analysis of the power spectrum.

### Histology

After completing the behavioral experiment, we passed a current of 50 μA for 45 s between the electrode tips under anesthesia with sodium pentobarbital (65 mg/kg i.p.) to confirm their location. Then, after several days, the rats were deeply anesthetized with an overdose of sodium pentobarbital (100 mg/kg i.p., Kyoritsu Seiyaku, Tokyo, Japan) and perfused transcardially with 0.9% saline, followed by 10% formalin. The brains were removed, stored in 10% formalin, and sectioned at 40 μm on a cryostat. The brain slices were mounted on glass slides and Nissl stained to examine the electrode placement under a microscope.

### Statistical analyses

Statistical analyses were conducted using statistical software (SPSS, Chicago, IL, USA). Data were expressed as mean ± standard error of the mean. We used a one-way repeated measures ANOVA or two-way repeated measures ANOVA. In addition, following the ANOVA, we used a paired *t*-test or pairwise comparison post-hoc test. Statistical significance was set at *p*<0.05.

## Results

During the recording sessions, per trial, the rats pressed the lever an average of 2.76 ± 0.33 times. In addition, the rats completed an average of 24.8 ± 2.7 trials per session. The average inter-trial interval was 44.2 ± 3.3 s. All the electrodes were placed in the dorsal hippocampus (Fig 2A) and the power spectrum calculated from the recorded LFP demonstrated a clear peak in the theta range when calculated over a conditioning session (Fig 2C). The rats showed a good performance throughout the recording sessions, during which the percentage of correct lever press was 86.4 ± 4.4%. To elucidate the relationships between the change in the hippocampal LFP and the behavioral states, we separately analyzed the LFPs during the following time-periods: (i) before the first lever press, (ii) during the initial lever presses, and (iii) after the termination of multiple lever presses.

### Spectrogram before the first lever press in the correct and incorrect trials

The spectrogram analysis of the hippocampal LFP before the first lever press revealed a gradual increase in the relative power of theta frequency range (6–9 Hz) in the correct trials (Fig 3Aa), as well as in the incorrect trials (Fig 3Ab). As shown, the increase started approximately 6 s before the first lever press. Analysis of the absolute power of LFP revealed a gradual increase in the relative theta power, which resulted from both an increase in the absolute power of theta frequency range and decrease in that of the other frequency ranges (Fig 3Ac and 3Ad).

To quantitatively compare the time course between the correct and incorrect trials, we calculated the power of 6–9 Hz of the theta-band. Fig 3Ba demonstrates the change in relative power of the theta band. We conducted a subsequent statistical analysis using the value of relative and absolute power as the dependent variable. A two-way repeated measures analysis of variance (ANOVA) did not show a significant interaction between the time and trial type (F _(46, 184)_ = 1.334, *p* > 0.05) and no significant effect of trial type (F _(1, 4)_ = 2.868, *p >* 0.05), but time (F _(46, 184)_ = 34.689, *p* < 10^−5^) had a significant effect on the relative power. The absolute power of theta-band also showed a similar change to that of the relative power (Fig 3Bb), confirming that theta power increased before the first lever press in the correct trials, as well as in the incorrect trials. Two-way ANOVA with repeated measures did not demonstrate a significant interaction between time and trial type (F _(46, 184)_ = 1.578, *p* > 0.05) and no significant effect of trial type (F _(1, 4)_ = 0.168, *p >* 0.05). However, there was a significant effect of time (F _(46, 184)_ = 3.766, *p* < 0.05) on the absolute power.

### Spectrogram before the first lever press in the right- and left-forelimb trials

We further analyzed the gradual increase in the relative and absolute theta powers before the first lever press by separating correct trials into those using the right or left forelimbs (Fig 4A and 4B). As shown, the increase was almost similar between the right-forelimb trials and left-forelimb trials. A two-way ANOVA with repeated measures applied to the relative theta power did not show a significant interaction between time and trial type (F _(46, 184)_ = 0.833, *p >* 0.05). Although the trial type had no effect (F _(1, 4)_ = 0.059, *p >* 0.05), time had a significant effect (F _(46, 184)_ = 34.491, *p* < 10^−5^) on the relative theta power. Similarly, in the absolute theta power, there was no significant interaction between time and trial type (F _(46, 184)_ = 1.341, *p >* 0.05) and no significant effect of trial type (F _(1, 4)_ = 0.047, *p >* 0.05). However, time had a significant effect (F _(46, 184)_ = 12.916, *p* < 10^−5^).

### Spectrogram after the termination of the multiple lever presses using the right or left-forelimb

Analysis of the spectrogram of hippocampal LFP after termination of the lever presses, during the period when the rats took the food and ate it, revealed that the elevated relative theta power associated with multiple lever press, gradually decreased to the basal level, 8–9 s after the final lever release, both in the right- and left-forelimb trials (Fig 5Aa and 5Ab, respectively). Analysis of the absolute power of LFP revealed that the gradual decrease in the relative theta power resulted from both the decrease in the absolute power of theta frequency range and increase of the other frequency ranges (Fig 5Ac and 5Ad).

To quantitatively compare the time course between the right- and the left-forelimb trials, we calculated the power of 6–9 Hz of the theta band. Fig 5Ba shows the change in relative power of the theta band. A two-way ANOVA with repeated measures did not show a significant interaction between time and trial type (F _(46,184)_ = 0.891, *p >* 0.05) and no significant effect of trial type (F _(1, 4)_ = 0.446, *p >* 0.05). Time, however, had a significant effect (F _(46, 184)_ = 16.063, *p* < 10^−5^) on the relative theta power. The absolute power of theta band also showed a similar, but somewhat earlier decrease, compared to that of the relative power (Fig 5Bb), confirming that the theta power decreased with the termination of multiple lever pressing, in the right-forelimb and left-forelimb trials. A two-way ANOVA with repeated measures did not demonstrate a significant interaction between time and trial type (F _(46, 184)_ = 0.943, *p >* 0.05). Trial type also had no significant effect (F _(1, 4)_ = 0.495, *p >* 0.05). Time, however, had a significant effect (F _(46, 184)_ = 1.629, *p* < 0.05) on the absolute power.

### Spectral difference between the correct and incorrect trials during the first and the second lever presses

The critical behavioral difference between the correct and incorrect trials was that although the incorrect trials during the first lever press was wrong, the second and subsequent lever presses were correct. All lever presses during the correct trails were correct. This means that the second lever press was the first correct lever press in incorrect trials. Therefore, we focused on the difference in the power spectrum during the first and second lever presses. Before examining the spectral difference, we checked behavioral difference by comparing the duration of the first and second lever presses, as well as the lever release between them (Fig 6A). The duration of lever release after the first incorrect lever press during the incorrect trials was longer than that after the first correct lever press during the correct trials. This suggested that the rat showed a pause period before they shifted from the incorrect choice to the correct choice. A two-way ANOVA with repeated measures did not show a significant interaction between trial type and lever press/release (F _(2, 8)_ = 4.393, *p* = 0.051) or a significant effect of lever press/release (F _(2, 8)_ = 4.419, *p* = 0.051). Trial type, however, had a significant effect (F _(1, 4)_ = 9.1594, *p <* 0.05).

Then, we investigated the change in power spectrum during the behavioral sequence from the first to the second lever press and found a difference between the correct and incorrect trials (Fig 6B). During the correct trials, the theta power increased after the release of the first correct lever and then decreased to the level of the first lever press (Fig 6Ba). In contrast, in the incorrect trials, although the theta power did not increase much after the release of the first incorrect lever press, it showed a large increase during the second correct lever press (Fig 6Bb). To quantitatively analyze the change in theta power during the behavioral sequence from the first to second lever press, we calculated the power of the 6–9 Hz of the theta band, normalized by the total power (1–30 Hz) of the first lever press in each trial type (Fig 6C). A one-way repeated measures ANOVA indicated a significant effect for both the correct trial (F _(2, 8)_ = 41.917, *p <* 10^−5^) and incorrect trial (F _(2, 8)_ = 5.106, *p* < 0.05). Additional analyses using paired-sample *t*-tests confirmed significant differences between the first press and first release (*p <* 0.01), first release and second press (*p <* 0.05), and the second press and first press (*p <* 0.05), during the correct trials. During the incorrect trials, however, a significant difference was only observed between the first press and second press (*p <* 0.05).

### Comparison of the change in theta frequency between the correct and incorrect trials and between the right- and left-forelimb trials

In addition to the theta power, we examined the change in theta frequency associated with multiple lever pressing. The weighted average frequency of theta oscillation started to increase approximately 6 s before the first lever press, both during the correct and incorrect trials (Fig 7A). A two-way ANOVA with repeated measures did not show a significant interaction between time and trial type (F _(9, 36)_ = 1.03, *p >* 0.05) and trial type had not significant effect (F _(1, 4)_ = 0.546, *p >* 0.05). Time, however, had a significant effect (F _(9, 36)_ = 6.965, *p* < 10^−5^). When the correct trials were divided to the right- and left-forelimb trials before the first lever press (Fig 7Ba), there was also no significant interaction between time and trial type (F _(9, 36)_ = 1.039, *p >* 0.05) and no significant effect of trial type (F _(1, 4)_ = 1.622, *p >* 0.05), but time had a significant effect (F _(9, 36)_ = 17.873, *p* < 10^−5^). This tendency of similarity in the change of dominant frequency between the right- and left-forelimb trials was almost the same for the data after the last lever press. The increased dominant frequency of theta oscillation was lowered after the termination of multiple lever pressing, both in the right- and left-forelimb correct trials (Fig 7Bb). It should be noted that the dominant theta frequency began to decrease before the release of the last lever and reached the basal level after 3–4 s, i.e., much earlier than the decrease in the relative theta power (Fig 5Ba), and seemed to occur in parallel to changes in absolute theta power (Fig 5Bb). A two-way ANOVA with repeated measures, applied to the data including those 2 s before the final lever release, did not show a significant interaction between time and trial type (F _(11, 44)_ = 0.599, *p* > 0.05). Time and trial type, however, had significant (F _(11, 44)_ = 5.848, *p* < 10^−5^) and marginal (F _(1, 4)_ = 7.889, *p* < 0.05 (= 0.048)), respectively. The pairwise comparison showed a significant difference between data at -2 s and at after 2 s (*p* < 0.05). However, there were no significant differences between data collected at 2 s and after 2 s, indicating that the dominant frequency returned to the basal level in approximately 2 s.

## Discussion

In the present study, we investigated the effects of different forelimb usage and lever choice on hippocampal oscillations during the preparatory phase for lever selection. We found that both the power and frequency of theta oscillation increased before the start of lever pressing, irrespective of whether lever choice and the paw used were correct, suggesting that these parameters might reflect a general preparatory process like an attentional process for multiple lever pressing and could be used as a triggering cue for the BMI to initiate processing of brain activities for controlling output devices.

### Prior increase in the power of hippocampal theta oscillation

Whishaw and Vanderwolf (1973) reported that the theta oscillation was observed during head turning and lever pressing, although it was much smaller than during large movements, such as running and jumping [25]. Buño and Velluti (1977) also reported that ongoing theta increased in amplitude and frequency at 1 s before and after the lever press, during the initial stage of sessions [26]. In addition, Sakimoto and Sakata reported a transient increase in hippocampal theta power after the stimulus, followed by a decrease before the lever press, during the reinforcement trials in simple discrimination and serial feature-negative tasks [27], as well as in simultaneous feature-negative patterning tasks [28]. This suggested that the hippocampal theta power reflected brain activity prior to the lever press. Consistent with this, we demonstrated that the theta power began to increase approximately 5–6 s before initial lever press. It remained elevated during a sequence of multiple lever presses and gradually decreased after the rats stopped pressing the lever to receive the food (Figs 3–5). Since theta power remained elevated during the sequence of lever on and off, within the multiple lever presses, the change in theta power may not be directly coupled to motor output, but rather could be involved in some cognitive processes. The long latency of 5–6 s from the initiation of theta power increase to the start of lever pressing, as well as the gradual decrease, lasting 5–6 s after the termination of lever pressing, also supports this view. Interestingly in a conditional visual discrimination task [29], the prior change in hippocampal theta power was different between the levers with distinct roles as follows: theta power increased 1 s before the center lever, which initiated a trial of the operant task, was pressed; however, it showed a large decrease before one of the two lateral levers for correct choice was pressed. In addition, the increase prior to the trial initiation was only observed during the initial stage of training, whereas the decrease before bar selection appeared even during the late well-learned stage. This lever selection between lateral bars was similar to that made in the initial lever press in our experiment, although the direction of change in theta power was contradictory. These results suggested that prior change in hippocampal theta power before lever press might be involved in several cognitive processes and depend on the task paradigm, as well as on the functional role of the lever assigned in the task.

We also demonstrated that this increase in theta power did not depend on the correctness of lever choice, suggesting that the prior increase in theta power might be associated with the start of lever pressing itself. This non-dependency on the correctness differed from findings in the negative patterning task, in which the relative theta power began to increase just before the correct lever press and diminished within 1 s in reinforced trials alone. This was not observed before the incorrect lever press in non-reinforced trials [30]. These results suggested that the power increase just before the lever press might depend on the correct cognition of the trial type in the negative pattering task. This is consistent with the findings during the decision-making period in four-arm radial maze, in which theta activity was elevated prior to correct choices, but not before incorrect ones [31]. In contrast to the present study, the increase in power that is initiated before the lever press might not be related to such cognitive events, since it was almost similar between correct and incorrect lever press, as well as between the right and left trials.

In addition, we found that the prior increase in theta power did not depend on the side of forelimb usage, suggesting that it did not directly couple to control of the contralateral forepaw movement. In the forelimb motor cortex, the theta power was significantly increased when the contralateral dominant hand was used during the food-reaching task [32]. Indirect coupling of the increase in hippocampal theta power to the contralateral forepaw control was also suggested by the long 6-s interval between the initiation of the theta increase and the start of lever pressing (Fig 4). Ohishi et al. (2003) reported that the evoked potential recorded in the contralateral forelimb motor cortex started 0.5–1.5 s before self-paced lever pressing and was eliminated by the ipsilateral hemicerebellectomy [33]. This suggested that pre-movement potential directly coupled to the control of contralateral forelimb started approximately 1 s before the lever press, which was much shorter than the start of increase in the hippocampal theta power in the present study.

Overall, the prior increase in the power of hippocampal theta oscillation in the present study may not be involved in the preprocessing of forelimb control or the cognition of trial type, but may represent the intention to start multiple lever presses.

### Prior increase in the hippocampal theta frequency

In addition to the change in theta power, the dominant theta frequency started to increase approximately 5–6 s before the first lever-press in the session, tended to decrease, but was maintained during multiple pressing, and then returned to the preparatory level after the termination of lever pressing (Fig 7). This suggested that there was no direct coupling to each lever on and off. This increase in frequency did not depend either on the correctness of the lever choice (Fig 7A) or the side of forelimb usage (Fig 7B). Therefore, the change in theta frequency almost paralleled that in theta power, both during the preparatory phase, as well as during the execution phase.

Simultaneous change in frequency and power of hippocampal theta oscillation, with a positive or negative correlation between them, has been reported in several types of behavior with large body movements [6, 25, 31], as well as in the lever pressing task [26]. For example, in the jump avoidance task, the theta frequency increased while the amplitude decreased, showing a negative correlation during the preparatory period of 0.5–1 s before the jump. During the execution of jumping, however, both the frequency and power increased rapidly and then decreased, showing a positive correlation [6]. In the lever pressing task, the amplitude and frequency of theta oscillation increased 1 s before and after the lever press, showing a positive correlation [26]. Thus, the frequency of theta oscillation might not always change in parallel with the power of theta oscillation, but depend on the type of movement and cognitive demand. This suggests that they have closely-coupled, different roles in preparing and executing motor behavior.

### Subsequent increase in the theta power after the first lever press

Although the prior increase in theta power was indistinguishable between the correct and incorrect trials, there was a significant difference in the change of theta power after the first lever choice. The theta power in the correct trials increased further after the first correct lever off and then decreased during the second correct lever on. The theta power in the incorrect trials, however, did not show a large increase after the first incorrect lever off, instead increasing greatly during the second “correct” lever on (Fig 6B and 6C). Therefore, the change in theta power after the start of multiple presses might reflect the change in cognition regarding the correctness of the lever press made by the animal. Overall, the increase in theta power might involve both attention- or decision-related gradual activities before the first lever press and transient cognition-related activities on the first correct choice. A similar two-phase increase in theta power was recorded from the medial prefrontal cortex and rostral anterior cingulate cortex in self-initiated hand-movement task with a waiting period, in which monkeys were trained to release the lever after holding it in a resting position for more than 6 s [34]. Theta power increased gradually 3–4 s before releasing the lever both in the rewarded and unrewarded trials and showed a second increase after the reward delivery in the rewarded trials or a decrease in the unrewarded trials. Although the second transient peak might involve the response to the reward, it was suggested that it includes the process of success/error judgment [34]. Since the rats were not rewarded until the robot came within their reach in the present study, the increase in theta power after the correct lever press might reflect success/error judgement at that point, rather than due to the rewarded response.

### Potential use of the prior and subsequent changes in hippocampal oscillation for BMI

There have been a lot of studies in humans that demonstrated the cortical potentials preceding self-initiated movements, which might be used for BMI. For example, it was shown that there were distinct patterns of cortical activity before the start of different self-initiated arm movements in healthy people as well as in patients suffering spinal cord injury, demonstrating that the intention of self-initiated limb movements could be detected using pre-movement cortical signals [9]. The intention to walk could also be detected by monitoring the pre-movement cortical potential in patients with stroke [10]. However, the change in cortical oscillation observed in these human studies was a decrease in the power of frequencies in the range of α and β bands (7–30 Hz), termed the event-related desynchronization, which began approximately 1 s before the movement and was sustained throughout the movement. In contrast, in the present animal study, the increase in hippocampal theta oscillation did not demonstrate laterality and started 5–6 s before the lever press, suggesting an intentional process that could be detected from brain oscillatory activities prior to cortical desynchronization. The present results would provide an additional possible source to improve the BMI, which could prepare the output device for incoming motor commands. In addition, the subsequent change in theta power after motor execution might also be useful to improve the BMI. If the post-execution increase in theta power, which contains information about the success/error judgement by the subject, is fed back to the BMI, it can act as an error-teaching signal to cause the BMI to learn to appropriately interpret cortical signals and produce correct motor commands. This would result in a gradual improvement of the BMI performance.

Although direct recording from the hippocampus would not be plausible in humans, the theta-band oscillation recorded from the frontal midline region of the scalp, which is known as FM theta, is a viable alternative. The human FM theta has been implicated in several mental operations, including working memory and attentional processes [11, 12]. Although the functional and pharmacological characteristics are not always the same between the hippocampal theta and the FM theta [12], the latter showed similar changes during the hand-movement task in monkeys [34] to those seen in the rat hippocampal theta in the present study. Therefore, the FM theta might be useful to improve the BMI by combining with other cortical activities.

## Conclusions

In the bimanual two-lever choice task with multiple presses, both the power and frequency of hippocampal theta oscillation increased 5–6 s before the first lever press, irrespective of whether the right or left forelimb was used and whether the first choice was correct. In addition, there was a significant increase in the theta power after the first correct lever press. These results suggested that the change in power and frequency of theta oscillation may reflect a preparatory process for lever pressing as well as cognitive processes after the lever press, both of which could be used to improve the BMI.

## Supporting information

S1 Fig Increase in the relative and absolute theta power before the first lever press in the right and left robot-presentation trial. (A) Dynamic power spectra before the first lever press in the right (a, c) or the left (b, d) presentation of the robot. The parameters used for the analysis were the same as in Fig 4. The relative power (a, b) and the normalized absolute power (c, d) of each frequency were calculated as in Fig 4. (B) Increase in the relative (a) and the normalized absolute (b) power of theta frequency band (6–9 Hz). The black and dotted lines indicate the data averaged over the each trial, and then over the rats. The shaded areas associated with the lines are standard error of the mean.
(TIFF)
