# Supplementary figures and images for: Change in hippocampal theta oscillation associated with multiple lever presses in a bimanual two-lever choice task for robot control in rats
Source: PLoS One. 2018 Feb 12;13(2):e0192593. doi: 10.1371/journal.pone.0192593 (PMC5809047; doi:10.1371/journal.pone.0192593)

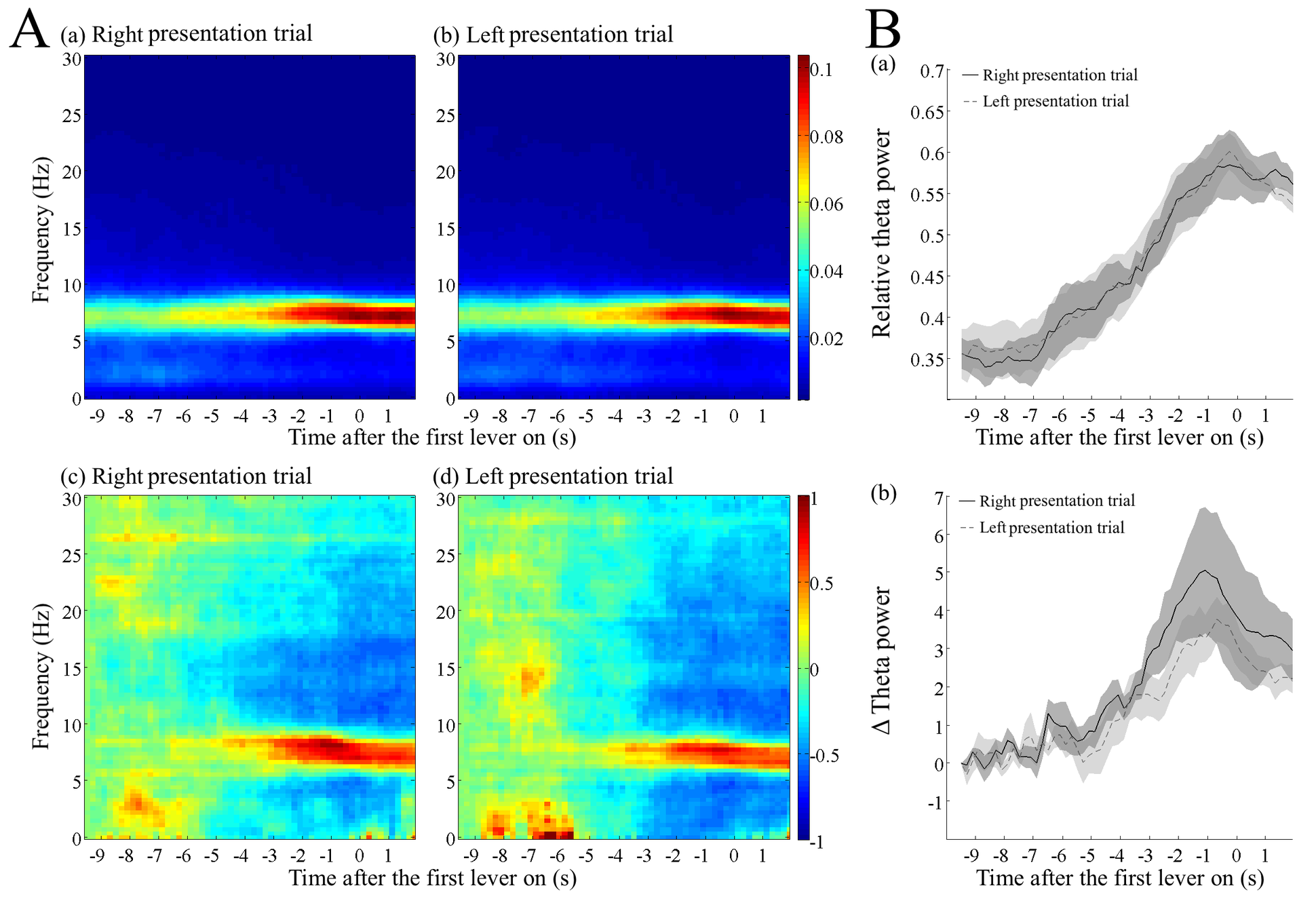

Supplement: S1 Fig — (A) Dynamic power spectra before the first lever press in the right (a, c) or the left (b, d) presentation of the robot. The parameters used for the analysis were the same as in Fig 4. The relative power (a, b) and the normalized absolute power (c, d) of each frequency were calculated as in Fig 4. (B) Increase in the relative (a) and the normalized absolute (b) power of theta frequency band (6–9 Hz). The black and dotted lines indicate the data averaged over the each trial, and then over the rats. The shaded areas associated with the lines are standard error of the mean. (TIFF) [file pone.0192593.s001.tiff]
